# Pamphlets—Spiritualism at the Church Congress

**Published:** 1882-01-20

**Authors:** 


### *PAMPHLETS, ETC.*

SPIRITUALISM AT THE CHURCH CONGRESS, by M. A. (Oxon), author of "Psychography," "Spirit Identity," "Higher Aspects of Spiritualism," etc., with advice and information for enquirers, and some additions by the American publishers. Price, 10 cents.—Religio-Philosophical Journal, Chicago.

A little work of 37 pages, containing a discussion in the Church Congress, an assembly of clergy and laity of the Established Church of England, at its late meeting at New Castle on Tyne.

This pamphlet will be likely to attract the attention of the clergy and intelligent Christian men who, whatever views they may have of the phenomena of spiritualism, are frequently insulted or disgusted at the very threshold of their investigations of this strange subject by the manifest hatred and bitter opposition toward the Christian Church which characterizes a large portion of the literature of the Spiritualists of this country.

Now that the truth of the phenomena has been recognized by certain prominent scientists in Germany, and that certain fair minded writers, as Watson and others, are presenting the subject in the aspect of a moral and Christian philosophy, we may expect that those who have heretofore refused to investigate it, even the clergy, will now be inclined to examine into its truth and merits. Aside from the moral aspect of the subject, it certainly presents questions of singular interest to the physiologist and the scientist. W.

